# Cooling Off and the Effects of Mandatory Breaks in Online Gambling: A Large-Scale Real-World Study

**DOI:** 10.1007/s11469-022-00996-7

**Published:** 2023-01-17

**Authors:** Niklas Hopfgartner, Michael Auer, Tiago Santos, Denis Helic, Mark D. Griffiths

**Affiliations:** 1 Institute of Interactive Systems and Data Science, Graz University of Technology, Sandgasse 36/III, 8010 Graz, Austria; 2 neccton GmbH, Davidgasse 5, 7052 Müllendorf, Austria; 3 International Gaming Research Unit, Psychology Department, Nottingham Trent University, 50 Shakespeare Street, Nottingham, NG1 4FQ UK

**Keywords:** Gambling, Responsible gambling, Responsible gambling tools, Problem gambling, Mandatory play break

## Abstract

The prevention of problematic online gambling behavior is a topic of major interest for regulators, the gambling industry, and researchers. Many gambling operators approach this issue by using responsible gambling tools. Among such tools, mandatory play breaks are used to interrupt long online gambling sessions, providing “cooling off” periods for players to take a reflective “time out”. The present study investigated the effects of mandatory play breaks in a large-scale experiment with 23,234 online gamblers engaging in more than 870,000,000 gambling transactions on *Norsk Tipping’s* gambling platform over a 1-month period. The gamblers were randomly assigned to several intervention groups with varying duration of mandatory play breaks and one control group with *Norsk Tipping’s* standard play break duration. More specifically, the study analyzed the relationship between the mandatory break received and the gambler’s acceptance of this tool, the interaction patterns with the tool, and how quickly they started to gamble again, as well as post-intervention effects on gambling behavior. Results showed that gamblers who were treated with longer mandatory breaks (i) tended to take longer voluntary breaks, and (ii) interacted more frequently with the tool (for instance, by clicking the “logout” button). Furthermore, gamblers appeared to accept longer mandatory play breaks. However, only a fraction of post-intervention effects remained, and mainly only for gamblers who received a substantial number of long mandatory play breaks. Overall, the present study provides actionable insights for both researchers and the gambling industry to improve the effectiveness of mandatory play breaks as a responsible gambling tool.

In moderation, gambling, and especially online gambling, is a fun and safe activity for most individuals (Francis et al., 2015; González-Roz et al., 2017). However, problematic gambling behavior significantly increases the risk of mood and anxiety disorders, personality disorders, and substance misuse (Petry et al., 2005), has a strong negative impact on the quality of life of family members (Wenzel et al., 2008), and may lead to gambling-related crimes, which typically consist of non-violent, income-generating offenses (Adolphe et al., 2019). Consequently, curbing and preventing problematic gambling behavior is not only a major concern for social workers, clinicians, and therapists, but also for gambling regulators, who mandate gambling operators to support individuals experiencing severe negative consequences of gambling (Bonello & Griffiths, 2017; Donati et al., 2014; Gooding & Tarrier, 2009). As such, this problem has also attracted a substantial interest of the research community (Auer et al., 2018; Blaszczynski et al., 2016; Hopfgartner, 2021b; Monaghan, 2009; Motka et al., 2018; Wohl et al., 2014; Wood & Wohl, 2015).

In an attempt to curb gambling problems, gambling operators have introduced (and researchers have evaluated) a range of responsible gambling (RG) tools, which inform gamblers about their behavior through personalized messages, protect them from overspending through limit-setting, or help them to limit their gambling time through voluntary self-exclusions and mandatory play breaks (Harris & Griffiths, 2017). Among these, tools that limit gambling time are particularly important as previous research showed that long uninterrupted gambling can induce psychologically rewarding, dissociative, and trance-like states, as well as provide an emotional or mental escape further reinforcing problematic gambling behavior (Weatherly et al., 2010). More specifically, an excessive engagement in such an escape activity might cause regular gamblers to gamble faster and more frequently (Harris & Griffiths, 2018), and also lead to gambling problems (Hing et al., 2016), which might be curbed by time limiting RG tools.

Currently, the efficacy of time-limiting tools in general and tools associated with mandatory play breaks is still an open question in the research community. Although it has been asserted that mandatory breaks facilitate “cooling off” periods and may end dissociative states (Griffiths, 2012), there are a few studies examining the effectiveness of mandatory play breaks one of which suggested that longer mandatory play breaks lead to unintended consequences such as increased cravings (Blaszczynski et al., 2016), and another which reported even higher stakes and longer gambling sessions immediately after the break (Auer et al., 2019). However, both studies suffered from some limitations. The experiment in the first study involved a simulated laboratory gambling task with 141 university students, which limits the generalization of the results to real gamblers (Gainsbury et al., 2014). In the second study, the authors analyzed real-world gamblers who received a mandatory play break after 1 h of continuous gambling. Lacking an experimental design, the authors compared all gamblers who received a mandatory break to matched gamblers who voluntarily stopped gambling a few minutes before the mandatory break. Therefore, the players who received a mandatory break might be more intense players compared to the players whose sessions were not interrupted, which might have led to a selection bias.

As previous research is inconclusive as to whether mandatory play breaks lead to “cooling off” or, on contrary, to accelerated gambling, Hopfgartner et al. (2021a) carried out a large-scale experiment with the Norwegian gambling operator *Norsk Tipping*. More specifically, *Norsk Tipping’s* mandatory play break RG tool was analyzed. At the time of the study, the tool comprises a mandatory break of 90 s for all gamblers who had played continuously for 60 min. When a break was triggered, the tool also showed a pop-up message informing players about how long they had been playing (i.e., 1 h) and the duration of the upcoming mandatory break. In a 1-month long experiment that comprised all *Norsk Tipping* players who received at least one play break during that period, players were randomly assigned to seven different groups. The authors tested three different durations of mandatory play breaks (i.e., 90 s, 5 min, and 15 min) with and without the inclusion of personalized feedback on the pop-up message of the mandatory play break, which informed the gamblers about their money lost/won during the last 24 h. These six groups also contained an additional “logout” button on the pop-up message. Finally, for comparison, one group was left unchanged and served as a control group. The results indicated that 15-min mandatory play breaks led to disproportionately longer voluntary play pauses compared to 5-min and 90-s mandatory play breaks and that personalized feedback during such play breaks appeared to have no additional effect on subsequent gambling. Furthermore, none of the different mandatory play breaks led to a change in the amount of money wagered once players began to gamble again.

The present study continued this line of research and controlled for pre-experimental behavior to better understand which gamblers benefited most from this experiment. Moreover, the interaction patterns with the pop-up message informing players about the mandatory play break were analyzed, as the results of the study by Hopfgartner et al. (2021a) suggested that players benefitted from an additional “logout” button on the pop-up message. Finally, it evaluated whether the experiment had any post-intervention effects by analyzing whether gamblers accepted longer mandatory play breaks and if they maintained longer additional voluntary play pauses once the experiment ended.

Consequently, the present study was designed to answer the following research questions (RQs): (i) how do different type of gamblers react to the various mandatory play breaks based on their pre-experimental behavior? (RQ1); (ii) does the experiment affect gamblers’ interaction patterns with the pop-up message of the mandatory play break? (RQ2); and (iii) do longer mandatory breaks cause gamblers to leave the platform, and do the effects of the different mandatory breaks persist after the experiment? (RQ3). This controlled and large-scale study of mandatory play breaks goes beyond the few previously published studies, which either lacked a controlled approach or comprised small-scale controlled laboratory studies. It was envisaged that the present study would provide actionable insights for researchers and the gambling industry on the duration, visual representation, effectiveness among different types of gamblers, and acceptance of mandatory play breaks as an RG tool.

## Method

The study comprised data from Norway’s national gambling operator *Norsk Tipping,* which offers both land-based (i.e., offline) and online gambling. In the digital channel, gamblers can play a choice of games including bingo, scratch-cards, slot games, and sports betting. *Norsk Tipping* requires each player to have a personalized account, which enables the operator to keep track of each individual’s gambling behavior. Moreover, *Norsk Tipping* has a monthly global loss limit of NOK 10,000 (approximately $1,000 US) on their digital channel, so that players cannot lose more money than that.

### Participants

The dataset contained 23,234 online players (61% male; mean age = 47.3 years) of *Norsk Tipping* who received at least one mandatory play break between 16 March 2020 and 31 July 2020. In total, it contained 879,286,736 entries. There were two types of entry. The first type of entry indicated a placed bet (e.g., a player bet NOK 10), and the second type of entry indicated a potential win associated with the previously placed bet (e.g., a player won NOK 15 as a result of the previous NOK 10 bet). In both cases, the entries contained information about the transaction amount (i.e., either stake or win) and the corresponding timestamp.

### Experimental Setup

Prior to the study, all players who gambled for a continuous period of approximately 60 min received a mandatory play break of 90 s. The mandatory play break showed up as a pop-up message containing a countdown, which informed the players that they could not gamble for the next 90 s. Between April 17 and May 21 (2020), the authors performed a controlled experiment where all online players who received at least one mandatory play break were randomly assigned to one out of seven intervention groups and stayed in this group until the end of the experiment. Since the results of the study by Hopfgartner et al. (2021a) indicated that personalized feedback had no additional effect on subsequent gambling, the three groups who received additional personalized feedback were merged to the respective intervention groups who did not receive such feedback. All experiments were repeated with and without the merged groups, and the results suggested that the inclusion of the groups who received personalized feedback in the present analyses yielded no significant differences. Therefore, gamblers were assigned to one of the following four intervention groups:

- *Control Group:* The Control Group received the same mandatory play break that already existed prior to the study. After 60 min of continuous gambling, a pop-up message appeared, which informed the players that they had been gambling for 60 min and that they would be receiving a 90-s play break. Furthermore, a countdown showed the remaining time of the mandatory play break and players could click a button, which redirected them to *Norsk Tipping’s* responsible gambling website.
- *Break 90 Group*,* Break 300 Group*,* or Break 900 Group:* These three conditions were identical to the Control Group, except for the duration of the mandatory play break and an additional “logout” button on the pop-up message. Players in the corresponding conditions received a pop-up message with either a 90-s, 300-s (5 min) or 900-s (15 min) play break after they had continuously gambled for 60 min. The only difference between the Break 90 Group and the Control Group was an additional “logout” button on the pop-up message.

Due to customer satisfaction concerns, approximately 60% of the gamblers were kept in the Control Group and therefore their gambling experience was not altered. All other gamblers were equally distributed to the remaining three conditions (i.e., approximately 13% of the remaining clientele per condition). After the experiment, the initial condition was restored, and all gamblers received the same mandatory play break as before the experiment.

**Fig. 1:**
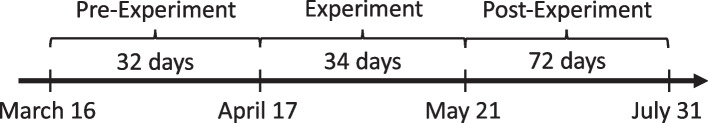
Illustration showing the temporal aspect of the study. The effects of mandatory play breaks on gambling behavior were analyzed between March 16 and July 31 (2020). Between April 17 and May 21, all players were randomly assigned to one of four experimental groups that received different mandatory play breaks. Before and after this period (i.e., pre- and post-experiment), all players received the default 90 s mandatory play break

Figure 1 shows the temporal aspect of the present study. During the whole period, gamblers received a total of 583,775 mandatory play breaks. The dataset also contained the beginning (i.e., a timestamp) and the duration of each mandatory play break. During the experimental period, the dataset also included information on how the gamblers interacted with the pop-up message, which informed them about the mandatory play break. More specifically, it contained information about whether gamblers (i) clicked on any of the buttons (i.e., “logout” or redirect to RG website), (ii) closed the web-browser, or (iii) did not interact with the pop-up at all.

**Fig. 2:**
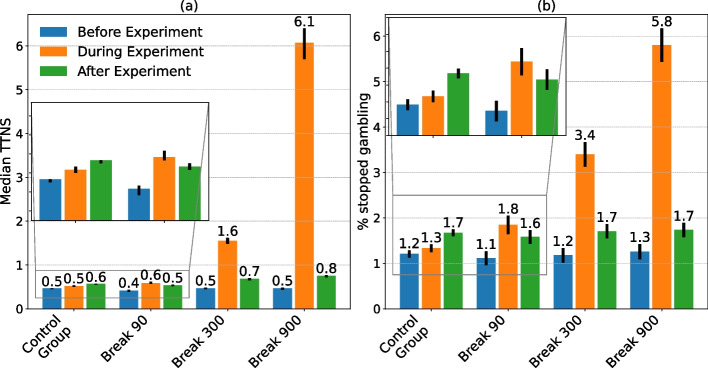
Bootstrapped distribution of the time to next session in minutes (**a,** TTNS), and the percentage of mandatory play breaks where players stopped gambling for that day (**b**). During the experiment gamblers significantly increased their TTNS as well as stopped gambling with longer mandatory play breaks. These effects significantly decreased again after the experiment

The authors only retained players in the dataset who had at least one mandatory play break before the experiment and one mandatory play break during the experiment. This enabled the authors to measure how players’ behavior changed during the experiment compared to before the experiment due to the different interventions. Therefore, the resulting dataset contained 12,510 players and 447,586 mandatory play breaks, with each player receiving at least two mandatory play breaks (i.e., one before and one during the experiment). As the initial study of the present experiment showed that the different mandatory play breaks did not affect gamblers’ amount bet after the break (Hopfgartner et al., 2021a), the present study focused on analyzing the time until players started to gamble again after a mandatory break (i.e., time to next session [TTNS]). Figure 2 provides an overview of the gambler’s overall TTNS (Fig. 2a), as well as percentage of mandatory play breaks where players stopped gambling for that day (Fig. 2b). To determine whether a player had stopped gambling for that day, the following two criteria were used: (i) the next session was not allowed to occur on the same day, and (ii) the TTNS must have been greater than or equal to 7 h. The threshold of 7 h was used to ensure that players had sufficient rest time (e.g., went to bed) if they have received a mandatory play break shortly before midnight. The 95% confidence intervals were bootstrapped using 10,000 repetitions.

### Statistical Analysis

The Bonferroni correction was used for all statistical tests, and the procedure on how the aforementioned research questions were addressed is explained below.

#### Hurried vs. Unhurried (RQ1)

The first research question focused on whether the experiment had different effects on players depending on their pre-experimental TTNS. For that reason, for each gambler the median TTNS was calculated for all mandatory play breaks prior to the experiment (i.e., between March 16 and April 16). The gamblers were then subsequently categorized into two groups according to the median of this measure across all players. Individuals above the median were referred to as *unhurried gamblers* (i.e., they took a pause before they started to gamble again), and individuals below the median were referred to as *hurried gamblers* (i.e., they started to gamble again quickly after the mandatory break). It was hypothesized that compared to unhurried gamblers, hurried gamblers would be more eager to play again in the intervention groups with longer mandatory play breaks (H_1_). To evaluate this hypothesis, the difference-in-differences method was applied to separate the actual intervention effects from concurrent (e.g., seasonal) effects that occurred even without the intervention (see seasonal effects in the Control Group in Fig. 2). Therefore, the difference between the intervention groups during and before the experiment was calculated and compared to the difference between the corresponding time periods in the Control Group. Therefore, by subtracting the difference between the control and the intervention groups (yielding the “difference-in-differences”), the changes that occur even without the intervention were separated out, providing better estimates of the intervention effects. Since gamblers’ TTNS in all periods consisted of repeated measures, the median TTNS per gambler and period was calculated to measure their overall behavior in each period. Using a regression-based difference-in-differences approach, the logarithm of the TTNS was used as the dependent variable, and as independent variables, the following two factors were used: *period*, which indicated whether the measurement of the TTNS was before or during the experiment, and *intervention,* which indicated a gamblers’ intervention group. The resulting regression model was as follows:

1 $$TTNS\sim {\beta }_{0}+{\beta }_{1} \;period+{\beta }_{2}\; intervention + {\beta }_{3}\; period:intervention$$

where *period:intervention* represents the interaction of *period* and *intervention*.

The logarithm of the TTNS was used as its untransformed form followed a power law distribution. Given the above difference-in-differences regression model, *ß*_1_ captured the seasonal effects between the pre-experimental and experimental period, whereas *ß*_2_ captured potential pre-experimental differences between the intervention groups. Finally, *ß*_3_ provided the estimates of the relative intervention effects. Instead of including a third factor in the regression model representing a gambler’s hurry group (i.e., hurried or unhurried), a simpler approach was used by conducting the difference-in-differences regression separately for these two groups.

In Table 1, descriptive statistics for the two hurry groups prior to the experiment are reported, including the median values of the gambling behavior in the 60 min before the mandatory play breaks. It shows that compared to unhurried gamblers, hurried gamblers staked significantly less money within 1 h prior the mandatory play break (*U*-test: *U* = 1,843,728,872, *p* < .001) and placed significantly less bets (*U*-test: *U* = 1,882,443,275, *p* < .001). However, compared to unhurried gamblers, hurried gamblers staked significantly more money per bet and received significantly more mandatory play breaks (*U*-test: *U* = 2,091,843,950, *p* < .001).

**Table 1:** Median values of the gambling behavior within 1 h before the mandatory play break for hurried and unhurried gamblers. Overall, hurried gamblers staked significantly less money within 1 h prior the mandatory play break (*U*-test: *p* < .001) and placed significantly less bets (*U*-test: *p* < .001) compared to unhurried gamblers. However, hurried gamblers staked significantly more amount per bet and received significantly more mandatory play breaks (*U*-test: *p* < .001) compared to unhurried gamblers

Hurry group	Hurried	Unhurried
Number of gamblers	6295	6214
Number of mandatory breaks	71,843	55,737
Q1 number of breaks per gambler	2	2
Median number of breaks per gambler	5	4
Q3 number of breaks per gambler	13	10
Median TTNS	0.17	1.40
Median amount bet 1 h prior to break	935	1 165
Median number of bets 1 h prior to break	309	339
Median amount per bet 1 h prior to break	5.83	5.00

#### Interaction with Pop-up (RQ2)

For the second research question, the study analyzed how gamblers interacted with the pop-up message during the experimental period, which informed them about the mandatory play break. A gambler could either click on any of the available buttons on the pop-up message, close the web-browser or wait until the mandatory play break was over. Although gamblers did not directly interact with the pop-up message when closing the web-browser, it was possible to distinguish whether gamblers closed the web-browser or waited until they could continue to gamble. If gamblers waited until they could continue to play, an event was recorded at the time the pop-up disappeared after the mandatory play break ended; otherwise, this event was missing. Due to technical issues, the dataset only contained the interaction behavior for 104,660 out of 124,142 mandatory play breaks during the experiment (84.3%).

It was hypothesized that gamblers who did not interact with the pop-up message at all (i.e., they waited until they could continue to play) would have a shorter TTNS (H_2_) and that gamblers who logged out would have a longer TTNS (H_3_). Moreover, it was hypothesized that the proportion of mandatory play breaks in which gamblers logged out would increase the longer the mandatory play break lasted (H_4_).

#### Post-Intervention Effects (RQ3)

The preliminary analysis in Fig. 2 shows that although all three intervention groups significantly decreased their TTNS after the experiment ended, the TTNS after the experiment was significantly higher in the Break 300 Group and the Break 900 Group compared to the Break 90 Group. Therefore, the present study further evaluated potential post-intervention effects on gamblers after the initial 90-s mandatory play break was restored. It was hypothesized that gamblers who received a high number of long mandatory play breaks (i.e., 15 min) during the experiment would experience a stronger post-intervention effect as compared to gamblers who only received a few 15-min mandatory play breaks (H_5_). To evaluate this hypothesis, the same difference-in-differences method as described in Eq. 1 was used. The only difference was that in this setup, the independent variable *period* indicated whether the median TTNS of a gambler was before or after the experiment. To allow for different intervention effect sizes between individuals who only received a few mandatory play breaks (e.g., one or two) and individuals who got many mandatory play breaks, gamblers were categorized into three equally sized groups (i.e., low, medium, and high) according to the number of mandatory play breaks (interventions) they had received during the experiment. Therefore, gamblers who received one to three mandatory play breaks were placed in the “low” group, gamblers with four to nine mandatory play breaks were placed in the “medium” group, and gamblers with more than nine mandatory play breaks were placed in the “high” group. Second, the authors evaluated if the different mandatory play breaks affected gambler retention. Retention was measured by calculating the proportion of gamblers which were still active after the experiment (i.e., placed at least one bet after the experiment).

### Ethics

This study was performed in line with the principles of the Declaration of Helsinki. Approval for the study was granted by the last author’s university ethics committee. Informed consent was not required for the present study because participants had already agreed to the platform’s terms of service, which included the use of their data for research purposes. By using the platform, participants gave their implicit consent to participate in the study.

## Results

### Intervention Effects for Hurried and Unhurried Gamblers

**Fig. 3:**
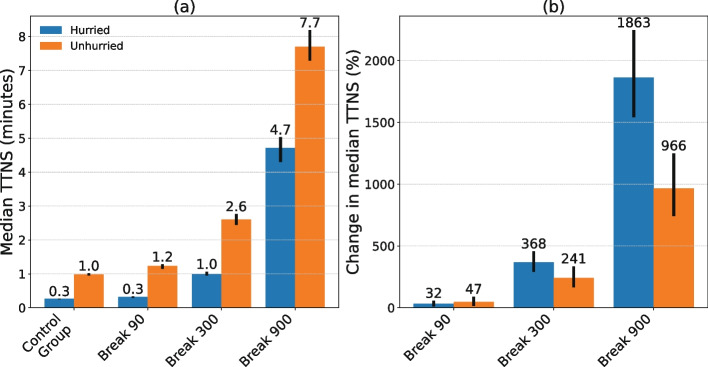
Absolute effects (**a**) and relative effects (**b**) of the mandatory play breaks on hurried and unhurried gamblers. The longer the mandatory play breaks, the stronger the intervention effect for hurried players compared to unhurried players

Figure 3a shows the bootstrapped median TTNS including 95% CI for both hurried and unhurried gamblers during the experiment. Similar to Fig. 2, it shows that throughout all intervention and hurry groups, the TTNS significantly increased the longer the mandatory play breaks lasted. Moreover, the absolute median TTNS of unhurried gamblers was significantly higher compared to hurried gamblers in all intervention groups. In Fig. 3b, the relative intervention effects (i.e., *ß*_3_) of the TTNS in each intervention and hurry group as defined in Eq. 1 are shown. More specifically, hurried gamblers in the 5-min mandatory play break condition increased their TTNS by 368%, whereas unhurried gamblers increased their TTNS by 241%. In the 15-min mandatory play break condition, there was a significant difference in the relative intervention effects between hurried (1863% increase) and unhurried gamblers (966% increase). In summary, these results indicate that hurried gamblers experienced a greater relative increase in their TTNS the longer the mandatory play break lasts compared to unhurried gamblers.

### Interaction Patterns

**Fig. 4:**
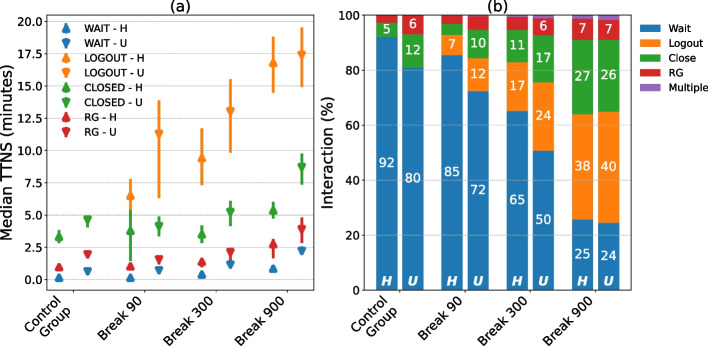
Interaction behavior with the pop-up message of the mandatory play break and its effect on the time to next session (**a**, TTNS), as well as the interaction distribution (**b**) for hurried (H) and unhurried (U) gamblers. Gamblers could choose to interact with the pop-up (i.e., by clicking on any of the available buttons or closing the entire web-browser) or wait until the mandatory play break was over without taking any action

Figure 4a shows the bootstrapped distribution of the TTNS depending on the gambler’s interaction with the pop-up message of the mandatory play break. It shows that throughout all intervention and hurry groups, players who did not interact with the pop-up at all (i.e., waited until the mandatory break was over) had the shortest TTNS. Gamblers who visited the RG website (by clicking the RG button) had a significantly longer TTNS than gamblers who did not interact with the pop-up. Moreover, gamblers who closed the web-browser had a significantly longer TTNS than gamblers who clicked on the RG button or waited until the mandatory play break was over. The longest TTNS was observed among gamblers who logged out via the respective button on the pop-up. Except for the Control Group (which did not have a “logout” button on the pop-up) and hurried gamblers in the Break 90 Group, logging out led to a significant longer TTNS than any other actions which gamblers could take.

Using the knowledge of Fig. 4a regarding how the interaction behavior affected the TTNS, the authors continued the analysis by inspecting the distribution of the interaction within each intervention group in Fig. 4b. This analysis showed that gamblers in the Control Group interacted the least with the pop-up (i.e., they waited until the pop-up message disappeared). In contrast, the highest proportion of interactions with the pop-up message was observed among gamblers in the intervention group with a 15-min mandatory play break (i.e., Break 900 Group). Overall, gamblers significantly increased the interaction with the pop-up message the longer the mandatory play break lasted (chi-square-test for comparisons between the Break 90 Group and the Break 300 Group: *χ*^2^ = 1545.6, *df* = 1, *p* < .001, and between the Break 300 Group and the Break 900 Group: *χ*^2^ = 3086.1, *df* = 1, *p* < .001), and that the presence of a “logout” button further increased the interaction (chi-square-test: *χ*^2^ = 468.6, *df* = 1, *p* < .001 for comparison between the Control Group and the Break 90 Group).

Moreover, within the intervention groups, hurried gamblers interacted with the pop-up significantly less compared to unhurried gamblers (chi-square-tests for Control Group: *χ*^2^ = 1733.9, *df* = 1, *p* < .001, Break 90 Group: *χ*^2^ = 379.4, *df* = 1, *p* < .001, and Break 300 Group: *χ*^2^ = 290.6, *df* = 1, *p* < .001). Only in the Break 900 (15-min) Group was there no significant difference between hurried and unhurried gamblers in the proportion of interaction with the pop-up message (chi-square-test: *χ*^2^ = 2.9, *df* = 1, *p* = .35). In sum, gamblers who logged out during the mandatory play break showed the strongest increase in the TTNS, and the longer the mandatory play break, the higher the proportion of gamblers who logged out and the smaller the differences between hurried and unhurried gamblers.

### Post-Intervention Effects

**Fig. 5:**
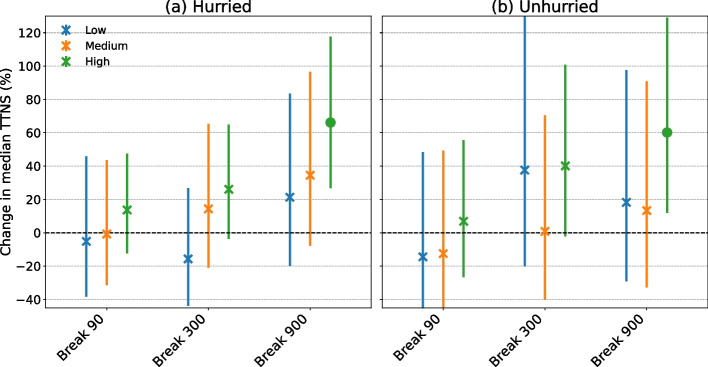
Long-term effects for hurried and unhurried gamblers measured as relative change (pre- and post-experiment) in the median TTNS estimated via difference-in-differences regressions across six subgroups of players

Figure 5a shows a positive association for hurried gamblers between the number of received interventions (i.e., mandatory play breaks) and potential post-intervention effects. However, only gamblers who received a high number (i.e., more than 9) of 15-min mandatory play breaks maintained a significant post-intervention effect, resulting in a 66% increase in TTNS. Furthermore, it showed a significant post-intervention effect (60% increase in TTNS) for unhurried gamblers, who received a high number of 15-min mandatory play breaks (Fig. 5b).

**Fig. 6:**
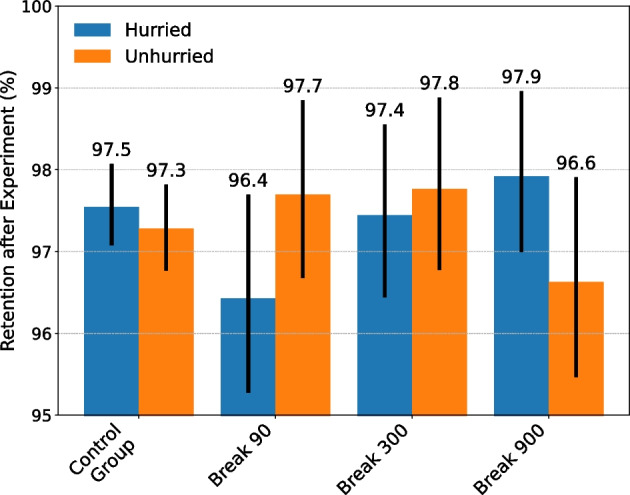
Bootstrapped proportion of gamblers who were still active (i.e., retention percentage) after the experiment. No significant differences in retention were observed between the groups

Figure 6 reports gambler retention including the bootstrapped 95% CI. In all intervention groups, approximately 97% of the gamblers were still active after the experiment. Therefore, the experiment had no significant effect on gambler retention. In sum, during the experiment, gamblers significantly increased their TTNS the longer the mandatory play break lasted, and most of this effect diminished after the experiment. Only players who received a high number of 15-min mandatory play breaks maintained a significant increase in the TTNS after the experiment. Overall, the findings indicate that there were no major long-term effects of the experiment.

## Discussion

In the present study, the effects of a large-scale experiment regarding mandatory play breaks among gamblers were investigated by analyzing player account-based data. The analysis resulted in four main findings. First, hurried gamblers (i.e., those who start to gamble again quickly after the mandatory break) experienced a greater increase in their TTNS the longer the mandatory play break lasted compared to unhurried gamblers. Second, logging out during a mandatory play break led to the strongest increase in TTNS, and a 15-min mandatory play break led to the highest proportion of gamblers logging out. Third, the experiment produced no considerable long-term effects, meaning that most players who received longer mandatory play breaks significantly changed their behavior back towards their pre-experimental level once the experiment ended. However, gamblers who received a high number (i.e., more than nine) of 15-min mandatory play breaks were able to maintain a small amount of this effect after the experiment. Finally, player retention remained the same across all intervention groups.

### Effects on Hurried and Unhurried Gamblers

The results indicated that longer mandatory play breaks exerted a stronger effect on hurried gamblers, leading to a stronger increase in their TTNS compared to unhurried gamblers. Surprisingly, this finding contradicted H_1_ that hurried gamblers would be more eager to play again because both gambling addiction research (and addiction research more generally) suggest that gamblers in cue-induced high-craving conditions show a steeper discount of delayed rewards as compared to low-craving conditions (Miedl et al., 2014), which may in turn affect the likelihood of relapse (Gorelick et al., 2012; Sheffer et al., 2012). Similarly, a study among individuals with internet addiction found that cue-induced craving was higher in this population compared to unaffected individuals (Niu et al., 2016). Assuming that the behavior of hurried gamblers might be more problematic compared to unhurried gamblers and that the measurement of TTNS negatively correlates with craving to play, these results are surprising in relation to prior work and warrant further investigation to reconcile the findings of the present study with those in the extant literature and to isolate potentially confounding factors.

### Interaction with the Pop-up Message

Consistent with H_2_ and H_3_, results showed that gamblers who logged out using the “logout” button on the pop-up message of the mandatory play break had the longest TTNS, whereas gamblers who waited until they could gamble again had the shortest TTNS. Assuming that players who log out may experience their behavior as more problematic than players who wait until they can gamble again, this result is consistent with existing literature suggesting that individuals who felt that they have lost control are more likely to self-exclude from gambling platforms (Motka et al., 2018). Previous work also suggests that individuals may temporarily deactivate their social media accounts during high-stress periods, when they feel that they spend too much time on it (Baumer et al., 2013). This supports the assumption that gamblers who log out might perceive their gambling behavior as more problematic (because they might feel that they spend too much time gambling) compared to gamblers who do not log out.

Furthermore, the results support H_4_, as they showed that the longer a mandatory play break lasted, the higher the proportion of gamblers who logged out. Qualitative research shows that one reason gamblers (especially problem gamblers) play is to achieve a dissociative state that distracts them from their problems (Griffiths et al., 2006; Weatherly et al., 2010; Wood & Griffiths, 2007). Therefore, longer mandatory play breaks could potentially help to end such dissociative states by promoting self-awareness (Monaghan, 2009), as more players log out.

### Post-Intervention Effects

First, most of the effect of a 5-min and 15-min mandatory play break on gamblers’ TTNS did not persist once the experiment ended and gamblers continued to get a 90-s mandatory play break. According to one study, there are four different pathways on how persistent intervention effects may arise, namely building psychological habits, changing what and how individuals think, changing future costs, and harnessing external reinforcement (Frey & Rogers, 2014). One reason for the lack of persistence of a 5-min or 15-min mandatory play break on gamblers’ TTNS could be the short duration of the experiment (i.e., approximately 1 month), where 50% of the gamblers received less than six mandatory play breaks. This supports H_5_ because the results of the second difference-in-differences regression model suggested that only gamblers who received more than nine 15-min mandatory play breaks were able to maintain a small amount of this effect. For habits to form (e.g., go for a walk once gamblers get interrupted by a mandatory play break), the environment must be stable while the environment-behavior association forms and must remain stable after the association is set (Wood et al., 2005). Since the environment changed after 1 month (i.e., gamblers started to receive 90-s mandatory play breaks again) this could have interrupted the process of forming a stable environment-behavior association too early. Furthermore, interrupting a gambler’s session without giving them information about their behavior in relation to other individuals might be another reason that gamblers did not change the thinking about their gambling.

Second, the experiment did not affect player retention, which is important because it showed that players also accepted receiving longer mandatory play breaks without problems. Therefore, operators can implement such interventions to remedy problematic behavior. If player retention decreased after the introduction of such an RG tool, it cannot be ruled out that gamblers would switch to another gambling operator with potentially less strict RG measures.

### Practical Implications

The present findings suggest multiple opportunities for the design of gambling interventions. First, future work could integrate normative feedback in mandatory play breaks (e.g., how many other gamblers played continuously for 1 h in the last 24 h), as well as include personalized information about how much money and time they could save if they reduced their gambling for a specific amount to facilitate players to rethink their own gambling behavior. Second, the results of the experiment suggest a permanent implementation of a longer mandatory play break (i.e., 15 min) after continuously gambling for 60 min to form a long-lasting positive effect. Third, longer mandatory play breaks could in turn promote self-awareness and help end dissociative states as more players make the conscious decision to log out. Finally, giving gamblers the possibility to log themselves out using a “logout” button on the pop-up message seems to additionally help players to stop gambling.

### Ethical Implications

Despite the potential to improve existing interventions as highlighted above, there are important ethical implications associated with the implementation of such quantitative analyses in practice. While understanding how different mandatory play breaks influence subsequent gambling behavior contributes to the development of better RG tools (which help gamblers to control their own behavior), it could also be (mis)used to intentionally implement less effective RG tools and pretend corporate social responsibility. However, the authors expect the ethical impact to be low, as the present study’s findings suggest that even short mandatory breaks improve matters. Overall, using the findings of the present study responsibly may not only be more ethical but also more sustainable, since both qualitative and quantitative research suggests that corporate social responsibility, particularly a commitment to responsible gambling programs, is strongly linked to customer satisfaction (Abarbanel et al., 2018; Auer et al., 2020; Kim et al., 2017) and that the adoption of such tools increases player loyalty to the gambling operator (Auer et al., 2021).

### Limitations

The unequal assignment of the control and intervention groups is one limitation of the present study. Therefore, some slight deviations in the population characteristics were observed. However, combining all the intervention groups to one group and comparing it to the Control Group yielded no significant differences in the population characteristics. Furthermore, the difference-in-differences method accounted for potential deviations between the intervention groups. Moreover, bingo games offer an in-game chat feature that allows players to talk with each other during the game. Consequently, some players may have noticed that they were being treated differently in terms of mandatory breaks, and it cannot be ruled out that they adjusted their behavior accordingly. However, only about 20% of all players received at least one mandatory play break during a bingo game, and only a proportion of these players may have noticed that they were treated differently. Therefore, the impact of this effect may be negligible.

Although the present study suggested that a 15-min mandatory play break had the strongest impact on players, it cannot be ruled out that gamblers switched to other gambling operators during this period to circumvent the intervention. However, as there was no change in player retention, it suggests that the interventions did not cause players to completely switch to gambling on another operator’s platform. Also, the only demographic data available were gender and age which meant that the impact of other factors that may have influenced the findings (e.g., ethnicity, educational background) could not be assessed. It should also be noted that the data were from only one gambling operator and therefore the findings cannot be necessarily generalized to all online gamblers. Therefore, replication studies with other gamblers playing with other operators are needed.

The COVID-19 pandemic may have also impacted the experiment, as research suggests that there was a significant decrease in online casino gaming intensity during this period (Auer & Griffiths, 2022a). However, a minority of gamblers reported increased gambling due to the pandemic, and this group experienced higher levels of gambling problems and changes in alcohol consumption (Brodeur et al., 2021). The present authors believe that, particularly for this vulnerable minority, mandatory play breaks may be an effective tool to reduce gambling-related harm.

## Conclusion

The present study is the first large-scale experimental study of mandatory play breaks among Norwegian gamblers. Significant effects in gambling behavior were found due to mandatory play breaks. Players took longer voluntary breaks from gambling the longer the mandatory play break lasted. This effect was even stronger for hurried gamblers, who usually started to gamble again quickly after a short mandatory play break. One explanation for this effect is that gamblers actively decided to stop gambling and therefore increased their interaction with the tool (e.g., by clicking the “logout” button on the pop-up message). The study also showed that gamblers accepted longer mandatory play breaks up to 15 min. However, most of the positive effects of such long mandatory play break diminished once the experiment ended and the default 90-s mandatory play break was restored.

The present study contributed to the mixed results of previous research on the efficacy of mandatory play breaks with a large-scale randomized controlled experiment. Knowing how the duration and design of mandatory play break influence gamblers’ behavior facilitates its improvement as an RG tool and supports the implementation of a 15-min mandatory play break after an hour of play. Overall, the findings of the present study provide actionable insights for researchers and the gambling industry to curb potentially problem gambling behavior.

Given that one recent study showed that a 60-min mandatory break after making ten monetary deposits in one day has positive effects on gamblers’ deposit and wagering behavior (Auer & Griffiths, 2022b), future studies could implement multiple triggers for mandatory play breaks (e.g., after several losses within a short period of time) and evaluate their efficacy. Evaluating different types of personalized feedback (e.g., normative feedback and potential money savings) during such mandatory play breaks is also an important avenue for the future research. Finally, evaluating the efficacy of mandatory play breaks across different types of gambling operators would shed more insight into whether this intervention is suitable for various gambling populations.

## Data Availability

The data are commercially sensitive and therefore not publicly available.
